# The Effect of an Irradiation-Induced Recombination Suppressing Inversion on the Genetic Stability and Biological Quality of a White Eye-Based *Aedes aegypti* Genetic Sexing Strain

**DOI:** 10.3390/insects13100946

**Published:** 2022-10-18

**Authors:** Muhammad Misbah-ul-Haq, Antonios A. Augustinos, Danilo O. Carvalho, Lucia Duran de la Fuente, Kostas Bourtzis

**Affiliations:** 1 Insect Pest Control Laboratory, Joint FAO/IAEA Centre of Nuclear Techniques in Food and Agriculture, 2444 Seibersdorf, Austria; 2 Nuclear Institute for Food and Agriculture, Peshawar 446, Pakistan

**Keywords:** sterile insect technique, vector control, dengue, Zika, chromosomal inversion

## Abstract

**Simple Summary:**

*Aedes aegypti* is a vector of viruses transmitting diseases such as dengue, chikungunya, Zika, and yellow fever. Current vector population control methods, based mainly on insecticides, are ineffective and raise concerns due to their negative impact on the environment and human health. In this frame, the sterile insect technique (SIT), which relies on the mass production and release of sterile males to reduce fertile crosses in the natural population, has recently gained increased interest as an environment-friendly, species-specific method. Sterile males are the ‘active’ component of SIT, however, for SIT to be effectively applied in mosquitoes, the efficient elimination of females from the male release batch is a prerequisite, since they are blood-feeders and transmit pathogenic microorganisms. To this end, developing and utilizing genetic sexing strains (GSS) that facilitate sex separation are highly desirable. However, GSS must be stable during rearing and competitive in the field to be considered for operational release projects. Here, we present our findings regarding the genetic stability and biological quality of an *Aedes aegypti* GSS that is based on the white eye mutation. Moreover, we discuss the effects of the incorporation of a recombination-suppressing inversion on the strain’s biological quality and on important fitness parameters.

**Abstract:**

*Aedes aegypti* is the primary vector of diseases such as dengue, chikungunya, Zika fever, and yellow fever. The sterile insect technique (SIT) has been proposed as a species-specific and environment-friendly tool for the suppression of mosquito vector populations as a major component of integrated vector management strategies. As female mosquitoes are blood-feeders and may transmit pathogenic microorganisms, mosquito SIT depends on the release of sterile males. Genetic sexing strains (GSS) can be used for the efficient and robust separation of males from females. Two *Ae. aegypti* GSS were recently developed by exploiting eye colour mutations, resulting in the Red-eye GSS (RGSS) and the White-eye GSS (WGSS). In this study, we compared two WGSS, with and without the chromosomal inversion 35 (Inv35), and evaluated their biological quality, including genetic stability. Our results suggest that the WGSS/Inv35 presents a low recombination rate and long-term genetic stability when recombinants are removed from the colony (filtering) and a slow accumulation of recombinants when they are not removed from the colony (non-filtering). The two strains were similar with respect to fecundity, pupal and adult recovery rates, pupation curve, and pupal weight. However, differences were detected in fertility, survival rate of females, and flight ability of males. The WGSS/Inv35 presented lower fertility, higher survival rate of females, and better flight ability of males compared to the WGSS.

## 1. Introduction

Vector-borne diseases are emerging worldwide at alarming rates due to processes such as global climatic change, land use, and socioeconomic changes, with more than 80% of the world’s population being at risk and mosquitoes being the largest contributor to it because most of these diseases are transmitted by them [1,2,3]. This global upsurge of mosquito-borne diseases is major concern in public health sectors, as these diseases are continually expanding their range to previously non-endemic areas and re-appearing in areas where they had decreased or had been eliminated for decades [2,4,5]. *Aedes aegypti* is the primary transmitter of viruses that cause dengue, chikungunya, and Zika and yellow fever, which are diseases with great impact on human health. This mosquito species usually breeds in urban environments and has successfully spread to most of the tropical and subtropical regions of the world [6,7,8,9].

As effective drugs and vaccines (except for yellow fever) are not available, population control is critical for combating mosquito-transmitted diseases, but also challenging as the currently available methods, which are largely based on insecticides, are not efficient and, at the same time, raise concerns for their impact on environment and the evolution of chemical resistance [10,11]. There is a dire need for developing alternative control methods such as the sterile insect technique (SIT) which is a birth control, environment-friendly, species-specific, non-polluting approach that is receiving renewed interest for the population suppression of mosquitoes [11,12,13,14,15]. Historically, SIT has successfully been used for the suppression, local eradication, containment, or prevention of the (re)establishment of populations of insect pests and disease vectors in different parts of world, always as a component of area-wide integrated pest management programmes [15,16]. SIT relies on periodic releases of a large number of sterile males, which, when they mate with wild females, produce no offspring, leading to the suppression of the target population.

In the Mediterranean fruit fly, *Ceratitis capitata* (medfly), it has been shown that the efficiency and cost-effectiveness of SIT applications can be significantly enhanced by the release of males only [17,18]. For mosquito SIT applications, efficient sex separation and removal of females prior to the release of males in the field is an important prerequisite, as female mosquitoes are vectors of major pathogens [19,20]. The quality evaluation of males is also an important step, since the sterile males have to compete with wild males for mating with wild females in order to induce sterility and suppress the target population [11,13,21].

Several methods have been developed for mosquito sex separation, however, most of them have been proven to be labour intensive and challenging to apply at a large scale with the required efficiency and accuracy [11,19]. Therefore, there are ongoing efforts focusing on novel sexing strategies and sorters, including the development of genetic sexing strains (GSS), which requires a selectable marker and the linkage of the wild type allele of this marker to the male determining region [19,22]. The development of two *Ae. aegypti* genetic sexing strains, Red-eye GSS and White-eye GSS, were recently reported using as selectable markers the *red eye* (*re*) and the *white eye* (*w*) colour genes, respectively [23].

Genetic recombination phenomena may affect the stability of GSS, as has been shown in the best characterized GSS, the *Ceratitis capitata* VIENNA 8 GSS, currently used for SIT programmes worldwide [24,25]. Genetic recombination is highly suppressed in males of fruit fly species, a phenomenon called achiasmy, supposed to have evolved to protect the integrity of heterogametic sex chromosomes [26,27]. Achiasmy has highly enhanced the genetic stability of medfly GSS, since male is the heterogametic sex and the restriction in males’ genetic recombination has been well documented [24,28,29]. In Aedes mosquitoes, however, recombination occurs in males and females at similar rates [30] (and references therein), and this puts at risk the genetic stability of the GSS, affecting both efficient rearing of the strains and efficient elimination of females from the male release batch. The introduction of elements such as chromosomal inversions have been proposed as a possibility to enhance the genetic ability of GSS [24,28,31]. Mass-rearing facilities using the medfly GSS (or other) have integrated a filtering rearing system (FRS) by maintaining a rather small colony under constant filtering (removal of recombinants), and going through a number of amplification cycles without filtering to provide sufficient numbers of males for release purposes, without compromising the initial colony, is also important for the long-term stability of the GSS used [24,25].

To address this concern, two chromosomal inversions, which are known suppressors of recombination, were developed on chromosome I of *Ae. aegypti*, Inv35 and Inv5 [30]. The Inv35 was introduced into the RGSS and the new strain, RGSS/Inv35, showed drastically reduced recombination rates [23]. In addition, the red-eye mutation and Inv35 were introgressed into seven different genomic backgrounds for the development of the respective GSS. In all cases, the sexing character of the strains was maintained and all novel GSSs presented significantly reduced genetic recombination rates in the presence of Inv35 [32,33]. In the present study, the long-term genetic stability and biological quality of White-eye GSS, with and without the Inv35, was assessed, as well as their suitability for SIT applications against *Ae. aegypti* populations.

## 2. Materials and Methods

**Mosquito strains:** Two strains were used in the present study, *Ae. aegypti* WGSS and WGSS/Inv35, whose establishment has been reported previously [23,30]. Both strains were maintained at the Insect Pest Control Laboratory at 27 ± 1 °C, 80% r.h., and L12:D12 photoperiod (including 1 h twilight), following established guidelines [34]. Adult mosquitoes were reared in 30 × 30 × 30 cm plastic cages (Bug-Dorm-1, MegaView Science Co., Taiwan) and were provided with 10% sucrose solution in urine plastic cups (200 mL) with cellulose sponge on the lid for feeding. Female adult mosquitoes were blood fed twice per week with mildly warm porcine blood filled in collagen casing. After 72 h of blood-feeding, plastic cups internally encircled with white strip of filter paper half dipped in water were placed inside cages for weekly egg collection. Egg cups were removed after 2 days, and egg papers were left to dry at least for one week before hatching in the room conditions described above.

**Genetic stability:** The genetic stability was assessed by estimating the recombination rate of the WGSS/Inv35, under filtering and non-filtering conditions, for up to 16 generations, as described in [23]. The expected phenotypes of this strain are black-eyed males and white-eye females, whereas the recombinant ones are white-eyed males and black-eyed females. At least 1500 pupae were screened every generation using a stereo microscope to determine the recombination rate (% number of recombinants/total number of individuals (expected + recombinant). In the case of the filtered colony, the recombinants were counted and discarded, whereas in the non-filtered colony, they were counted and then reintroduced in the colony. The population size of both colonies (“filtered” and “non-filtered”) was at least 1500 individuals in every generation.

**Quality control analysis:** The impact of Inv35 was assessed by investigating the following quality control parameters, in a comparative way, in WGSS and WGSS/Inv35:

*Female fecundity*: For recording the fecundity (egg production), 50 males and 50 females were released together in adult cages (BugDorm-1 rearing cage 30 × 30 × 30 cm) and allowed to mate for 2–3 days. Mated females were blood fed and then shifted to separate small cages (BugDorm-4S1515 with 15 × 15 × 15 cm). On the next day, previously fed females were again blood fed to confirm full engorgement. Three replicate small cages were set up each consisting of 10 gravid females. Egg collection was done as mentioned above. The eggs were recorded and fecundity was estimated as the average number of eggs per female per replicate cage. The same process was followed for both the first and the second gonotrophic cycle. All egg papers after counting were kept in plastic trays to dry at 27 °C for a week. Counted eggs of each replicate were used for downstream experiments, i.e., to determine the fertility.

*Fertility*: All counted eggs from the fecundity test (from all replicates of both gonotrophic cycles) were hatched in airtight glass jars (500 mL) having low dissolved oxygen water and 2–4 drops of larval diet. The jars were kept for egg hatching in an incubator on constant temperature of 27 °C for 24 h. First instar larvae (L1) were then shifted to white plastic trays and counted by aspirating them with a plastic Pasteur pipette. The fertility was determined as number of L1/total number of eggs × 100.

*Pupal weight*: Pupae were first dried for 20 min at 27 °C and then weighted in batches of ten. Three replicates were performed per sex per strain using at least 24 h old pupae.

*Pupation time*: The pupation time is defined as the time required from egg hatching to pupation. The pupation curve was determined by counting the number of pupae daily (approximately at the same time each day) for all replicates per sex per strain.

*Recovery rates during development*: All counted L1 larvae from three replicates of the fertility test were shifted to white plastic trays and provided with 15 mL of larval diet as described previously [33]. The L1 larvae that survived up to pupal and adult stage were counted to estimate the respective recovery rates during development (from egg to adult).

*Adult sex ratio*: Adult sex ratio (% number of males/total number of adults) was determined by counting all males and females at the adult stage for all replicates per strain.

*Survival rate*: A total of 50 young (0–1 day old) virgin males and females of each strain and sex were put in separate cages and were provided food (10% sucrose solution) and water. Dead mosquitoes were counted for 30 days on a daily basis. Three replicates were performed per sex per strain.

*Flight ability*: Overall, 100 males per replicate with 4–5 days old were used to obtain the escape rate of each strain. For this parameter evaluation, males were released in Flight Test Devices (FTD), and the test was performed as described previously [21]. The percentage of successful male mosquitoes that managed to escape from the flight tubes within two hours represents the escape rate.

**Statistical analysis:** Statistical analysis was performed using R language 4.0.2—“Bird Hippie” with RStudio environment—version 1.3.1073. Data frequency distribution and its point distribution of the quantile-quantile plot were used to evaluate data normality and to confirm whether they are parametric or not. The alpha < 0.05 was considered as statistically significant for all analyses carried out. The Kaplan-Meier and Log-rank test were employed to perform the survival analysis [35,36,37]. Additional packages used in this analysis are presented in Supplementary File S1.

## 3. Results

### 3.1. Genetic Stability

**Figure 1:**
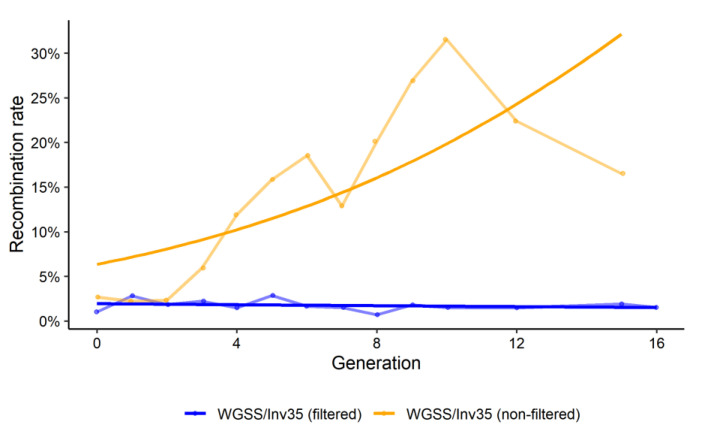
Recombination rate of the WGSS/Inv35 strain over 16 generations under filtering and non-filtering conditions. Statistical analysis was carried out using a GLM with binomial distribution and indicated significant differences between filtered and non-filtered conditions. The lighter lines indicate the means of the experimental data, whereas the darker lines indicate the generalized linear model fitted curve for the binomial distribution.

The recombination rate was recorded in the WGSS/Inv35 strain up to generation 16, thus expanding the analysis of our previous study, in which it had only been assessed up to the 4th generation [23]. In accordance with our previous results, the incorporation of the inversion (Inv35) greatly enhanced the genetic stability of the GSS (Figure 1). The mean recombination rate was estimated to 1.74% in filtered conditions compared to a mean of 14.6% in non-filtered conditions during the 16 generations of monitoring (Binomial GLM: deviance 25.69, df = 1, *p* = 3.12^−5^).

The frequency of recombinant males was higher in males than females under non-filtering conditions (Binomial GLM: deviance 9.165, *p* = 5.82^−3^), whereas no difference was observed under filtering conditions (Binomial GLM: deviance 0.777, *p* = 0.386).

### 3.2. Quality Control Analysis

**Figure 2:**
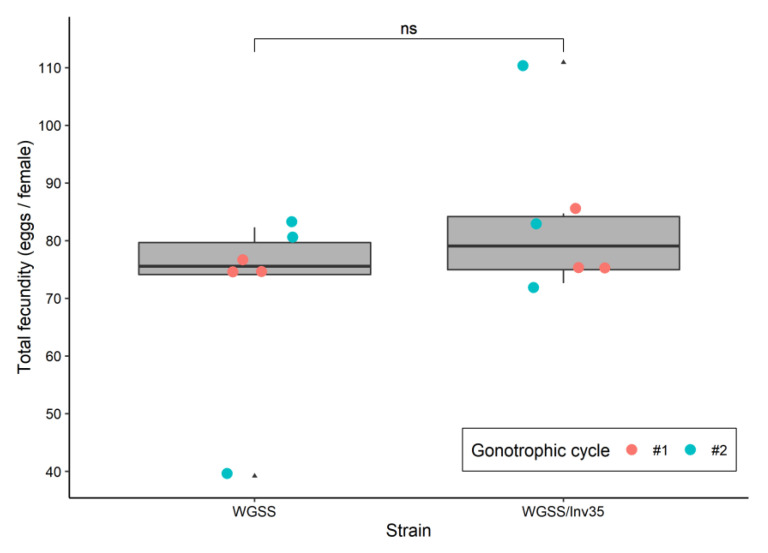
Total fecundity assay (number of eggs per female) of WGSS and the White-eye WGSS/Inv35. Three replicates were performed per gonotrophic cycle per strain. Statistical analysis was carried out using a GLM with Poisson distribution. The whiskers indicate the variability outside the upper and lower quartiles (represented as the upper and lower boxes, respectively). The thick horizontal line represents the median and the triangles indicate a possible outlier. Significance symbol: ‘ns’ for ‘not significant’.

*Fecundity*: The total fecundity (the first two gonotrophic cycles) did not differ between the *Ae. aegypti* WGSS and WGSS/Inv35, with the mean number of eggs per female being 71.2 (SEM 6.5) and 83.5 (SEM 5.8), respectively (Poisson GLM: deviance 1.734, *p* = 0.224) (Figure 2). The number of eggs produced per female during the first or the second gonotrophic cycle also did not differ between the two strains (Poisson GLM: deviance 0.616, *p* = 0.451) (Figure 2).

**Figure 3:**
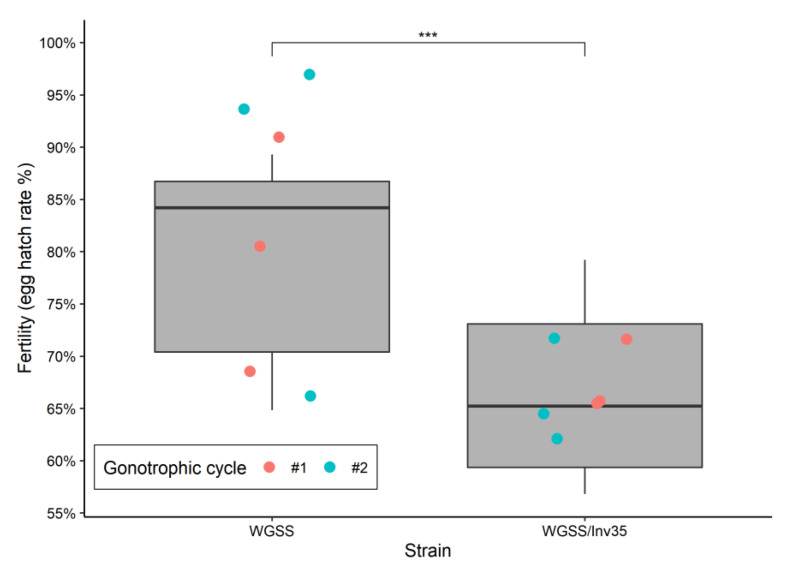
Fertility (100% × no. L1 larvae/total no. eggs) of WGSS and WGSS/Inv35. Three replicates were performed per gonotrophic cycle per strain. Statistical analysis was carried out using a GLM with binomial distribution and showed significant difference between the strains. The whiskers indicate the variability outside the upper and lower quartiles (represented as the upper and lower boxes, respectively). The thick horizontal line represents the median and the triangles indicate a possible outlier. Significance symbol: ‘***’ *p* value lower than 0.001.

*Fertility*: As shown in Figure 3, the WGSS and WGSS/Inv35 strains differ significantly regarding the fertility, 79.3% (SEM 4.44) and 66.6% (SEM 3.75), respectively (Binomial GLM—Tukey Contrasts: −9.162, *p* = 2.0^−16^). There was no difference for both strains as regards the fertility recorded in each of the first two gonotrophic cycles (Binomial GLM—Tukey Contrasts 1.264, *p* = 0.206) (Figure 3).

**Figure 4:**
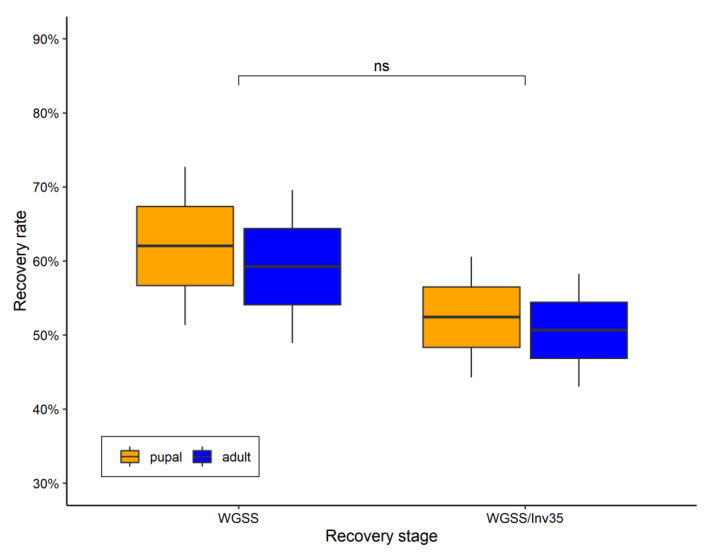
Recovery rates during different developmental stages for WGSS and the WGSS/Inv35. Three replicates were performed per strain. Statistical analysis was carried out using a GLM with binomial distribution. The whiskers indicate the variability outside the upper and lower quartiles (represented as the upper and lower boxes, respectively). The thick horizontal line represents the median.

**Figure 5:**
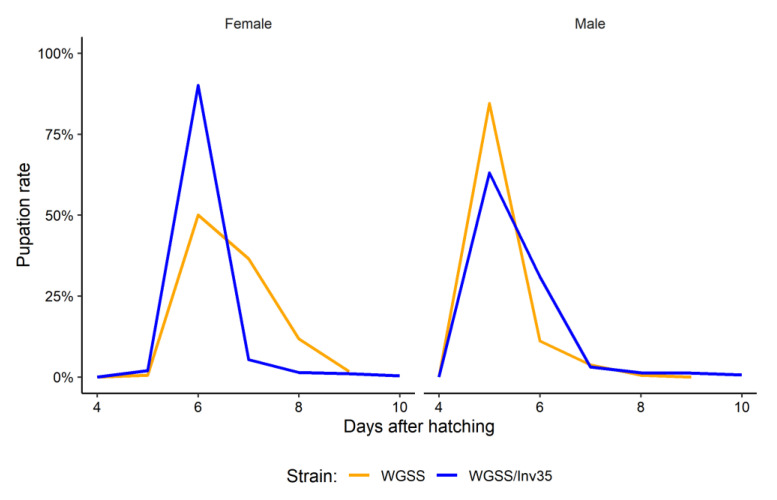
Pupation curves of females and males of WGSS and WGSS/Inv35. Three replicates were performed per strain. Statistical analysis was carried out using a GLM with binomial distribution.

*Recovery rates during development*: The WGSS and the WGSS/Inv35 strains did not differ significantly regarding the pupal recovery rates (Binomial GLM—Tukey Contrasts: −0.393, *p* = 8.46^−1^) or the adult recovery rates between the two strains (Binomial GLM—Tukey Contrasts −0.348, *p* = 8.63^−1^) (Figure 4). There was also no statistically significant difference in the sex ratio: 0.535 for WGSS and 0.518 for WGSS/Inv35 (Poisson GLM: deviance 0.28, *p* = 0.625). In addition, there was no difference between the two strains in respect to the pupation curve (Binomial GLM: deviance 0.171, *p* = 0.680). In both strains, pupation started earlier in males than females. The peak for males was observed 5 days after the hatching, whereas it was 6 days for females (Figure 5).

**Figure 6:**
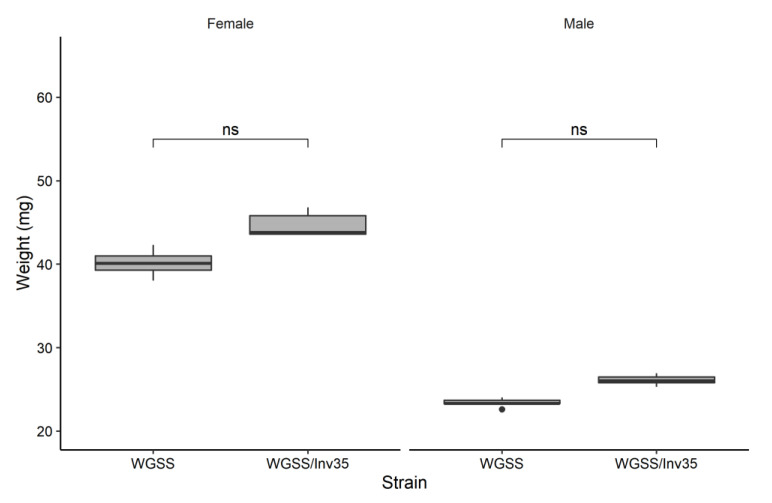
Weight per 10 male and female pupae of WGSS and WGSS/Inv35 strains. Three replicates were performed per sex per strain. Statistical analysis was carried out using a GLM with Poisson distribution. The whiskers indicate the variability outside the upper and lower quartiles (represented as the upper and lower boxes, respectively). The thick horizontal line represents the median, and the dot indicates a possible outlier. Significance symbol: ‘ns’ for ‘not significant’.

*Pupal weight*: No statistically significant difference was observed between males or females in both *Ae. aegypti* WGSS and WGSS/Inv35 regarding pupal weight, with females being heavier (approx. 42.4 mg per 10 pupae—Poisson GLM: Tukey contrasts 0.109, *p* = 0.966) than males (approx. 24.7 mg per 10 pupae—Poisson GLM: Tukey contrasts 0.11, *p* = 9.78^−01^) (Figure 6).

**Figure 7:**
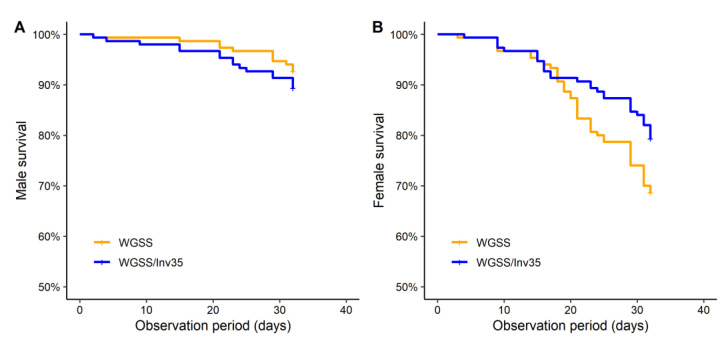
Representative Kaplan–Meier survival curves are shown for WGSS and WGSS/Inv35 males (**A**) and females (**B**). Three replicates were performed per strain. Statistical analysis was carried out using a log-rank test and showed significant difference between the strains.

*Survival rate*: Our analysis showed that t WGSS and WGSS/Inv35 strains did not differ regarding male survival rate during the first 30 days post emergence (*χ*^2^ = 1.1, d.f. = 1, *p* = 0.3). However, there was a statistically significant difference in the survival rate of females between the two strains, with higher survival rates recorded for WGSS/Inv35 females than WGSS females during the same 30-days period (*χ*^2^ = 4.4, d.f. = 1, *p* = 0.04) (Figure 7).

**Figure 8:**
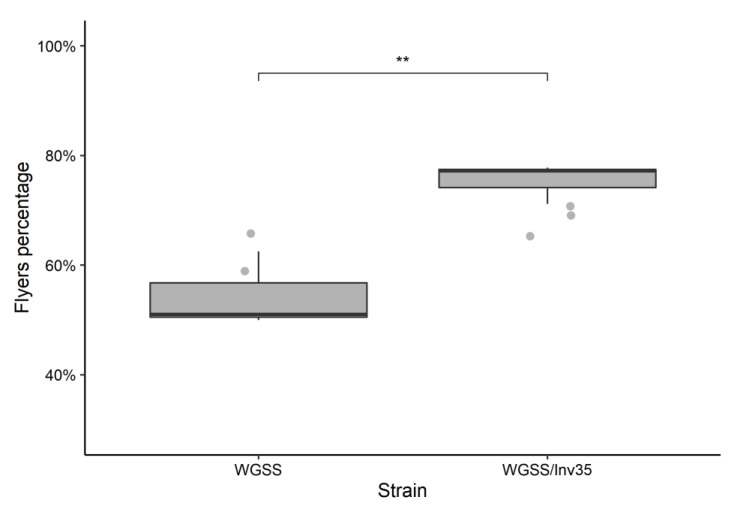
Flight ability of WGSS and WGSS/Inv35 males. Three replicates were performed per strain. Statistical analysis was carried out using a GLM with binomial distribution and showed significant difference between the strains. The whiskers indicate the variability outside the upper and lower quartiles (represented as the upper and lower boxes, respectively). The thick horizontal line represents the median. Significance symbol: ‘**’ for *p* value less than 0.01.

*Flight ability*: In respect to the flight ability, WGSS and WGSS/Inv35 strains differed significantly, with the latter presenting higher average percentage of flyers, 75% vs. 55% (Binomial GLM: deviance 21.19, df = 1, *p* = 0.01; Figure 8).

## 4. Discussion

SIT, as a component of area-wide integrated pest management programmes (AW-IPM), has been used for the population control of insect pests and disease vectors worldwide [15,16]. It has been shown that, in the availability of an efficient genetic sexing strategy, the efficacy and cost-effectiveness of SIT is significantly enhanced by releasing only males, which can be achieved through the development and use of genetic sexing strains (GSS) [17,18,19]. However, especially for mosquitos, the elimination of females from the male release batch is mandatory. The present study performed a comparative analysis of the *Ae. aegypti* WGSS and GSS/Inv35 strains. Our results showed that: (a) genetic recombination can be drastically reduced by Inv35, thus enhancing the genetic stability of the GSS; and (b) WGSS and WGSS/Inv35 differ in some of the quality control parameters studied.

We previously reported the development of two GSS using as selectable trait, the eye-color and the mutations red-eye and white-eye [23,30]. As genetic recombination can affect the stability of a GSS, we isolated an irradiation-induced chromosomal inversion, Inv35, which was subsequently incorporated in the GSS, thus significantly suppressing their recombination rates [23,30]. Moreover, RGSS and RGSS/Inv35 were introgressed into different genomic backgrounds, and it was shown that the sexing properties of the GSS remained fully functional, as well as the recombination-suppressing activity of Inv35, for many generations [32,33]. The results of these studies indicated that the recombination frequency between the white-eye and M loci can be reduced by Inv35, as monitored for a period of 16 generations, thus significantly improving the stability of the genetic sexing strain, particularly under filtering conditions. It is important to note that in medfly’s VIENNA GSS, strains’ collapse under non-filtering conditions is mainly attributed to the accumulation of recombinant females, due to their increased fitness compared to the non-recombinant ones, a phenomenon directly correlated to the reduced fitness of the *tsl* mutation itself (or closely linked loci) [24]. However, in the present case, although the white-eye mutation is associated with reduced fitness, strain collapse seems to be attributed mainly to the accumulation of male recombinants. This requires further investigation; however, it is likely that these recombinants possess biological properties that make them fitter than the non-recombinant ones.

Quality assessment is a prerequisite prior to the use of any strain in field applications [38]. The initially constructed RGSS and WGSS were comparatively assessed, and it was shown that the RGSS was a better performing strain in several quality control parameters and was selected for further evaluation including the incorporation of Inv35 [23,32]. It was recently shown that the introgression of the red-eye selectable marker and Inv35 in Pakistani genetic background did not affect the sexing properties and genetic stability, but it did have an impact, positive or negative, on several quality control parameters [33]. It is also known that chromosomal inversion may also affect fitness, positively or negatively [39,40,41]. To further elaborate these findings, the biological quality of the White-eye GSS was assessed, with and without the inversion Inv35. We focused both on rearing efficiency important characteristics (genetic stability, fecundity, fertility, pupal and adult recovery rates, and pupal weight), and traits important for the field performance of males (survival rate and flight ability) to gain insight into the possible impact of Inv35.

Productivity is an important parameter in mass rearing and SIT applications, as sufficient numbers of males need to be produced for irradiation-induced sterilization and subsequent releases over the targeted area for the population suppression of an insect pest species or disease vector. Therefore, fecundity, fertility, pupal and adult recovery rates, as well as sex ratio were evaluated in the WGSS and WGSS/Inv35 strains. Fertility was reduced in the WGSS/Inv35; however, the two strains did not differ in any of the other traits.

Our study also showed that the two strains did not differ regarding their pupation curve and pupal weight. This is an important finding because both protandry and pupal size are currently used as tools for separating males and females to achieve male-only releases for SIT applications [11,19]. A sex-specific difference was observed between WGSS and WGSS/Inv35 regarding survival rates. No statistically significant difference was detected in males, whereas there was a significant difference between the females of the two strains, with higher survival rates being observed in the WGSS/Inv35 strain. Interestingly, WGSS/Inv35 also presented higher numbers of male flyers compared to WGSS. This is an important observation, as flight ability is one of the key factors determining the quality of males released in the field for SIT applications.

## 5. Conclusions

Taken together, the data presented in this study suggest that, in several biological traits tested, there are no major differences between WGSS and WGSS/Inv35. In addition, the presence of Inv35 enhances genetic stability and perhaps contributes to the better flying ability of the males.

Strains may exhibit different restrictions when mass-reared for release purposes. Many putative bottlenecks do not manifest in rather relaxed laboratory conditions. Among other important characters, male mating competitiveness should be thoroughly assessed before any of these strains are considered in SIT field applications. Additional parameters that can be compromised by mass-rearing and are important for males to be released, such as dispersal capacity, survival, and competitiveness, must be studied exhaustively in larger arenas (field cages and semi-field conditions) to simulate (as possible) field performance.

## Data Availability

The original contributions presented in the study are included in the article/Supplementary Material, further inquiries can be directed to the corresponding authors.

## Supplementary Materials

The following supporting information can be downloaded at: https://www.mdpi.com/article/10.3390/insects13100946/s1.
