# Supplementary material for: The Effect of an Irradiation-Induced Recombination Suppressing Inversion on the Genetic Stability and Biological Quality of a White Eye-Based Aedes aegypti Genetic Sexing Strain
Source: Insects. 2022 Oct 18;13(10):946. doi: 10.3390/insects13100946 (PMC9604213; doi:10.3390/insects13100946)
Supplement: Supplementary file 1 [file insects-13-00946-s001.zip › insects-1876209-Supplementary File S1.pdf]

Supplementary Material - Statistical Report

Genetic Stability

Assessing the recombination rate for White-eye GSS/Inv35 over several generations under filtering and non-filtering conditions.

Table 01. Recombination rate in each generation for each filtering status

Summary recombinant rate table for filtering status

Filtering status	Generation	Recombination rate (%)
filtered	0	1.011
filtered	1	2.804
filtered	2	1.847
filtered	3	2.234
filtered	4	1.493
filtered	5	2.872
filtered	6	1.650
filtered	7	1.523
filtered	8	0.701
filtered	9	1.792
filtered	10	1.516
filtered	12	1.491
filtered	15	1.900
filtered	16	1.529
non-filtered	0	2.692
non-filtered	1	2.220
non-filtered	2	2.340
non-filtered	3	5.941
non-filtered	4	11.902
non-filtered	5	15.849
non-filtered	6	18.524
non-filtered	7	12.886
non-filtered	8	20.116
non-filtered	9	26.912

Filtering status	Generation	Recombination rate (%)
non-filtered	10	31.532
non-filtered	12	22.392
non-filtered	15	16.509

Generalized Linear Model

GLM to indicate differences among the variable *generation*, in relation to the recombination rate. Strain GLM summary:

```
##                  Df Sum Sq Mean Sq F value   Pr(>F)    
## filtering_status  1 0.1115 0.11150   25.69 3.12e-05 ***
## Residuals        25 0.1085 0.00434                     
## ---
## Signif. codes:  0 '***' 0.001 '**' 0.01 '*' 0.05 '.' 0.1 ' ' 1

##             Df    Sum Sq   Mean Sq F value Pr(>F)
## stage        1 0.0000521 5.208e-05   0.777  0.386
## Residuals   26 0.0017438 6.707e-05

##             Df Sum Sq Mean Sq F value  Pr(>F)   
## stage        1 0.1350 0.13495   9.165 0.00582 **
## Residuals   24 0.3534 0.01473                   
## ---
## Signif. codes:  0 '***' 0.001 '**' 0.01 '*' 0.05 '.' 0.1 ' ' 1
```

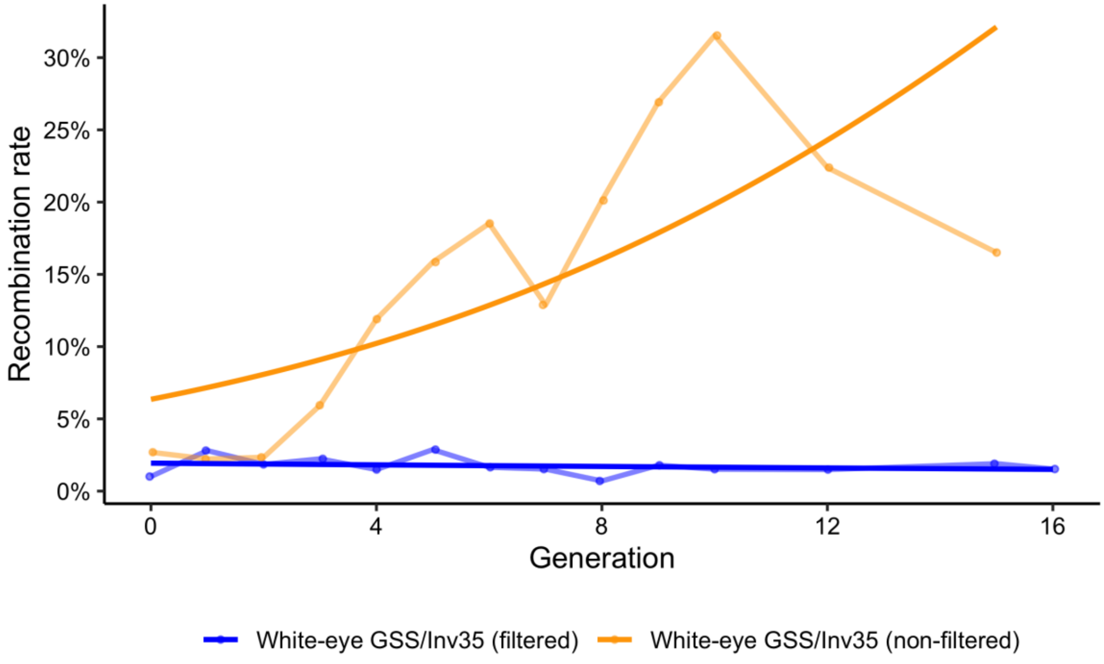

Figure 1. *Recombination rate of the White-eye GSS/Inv35 strain over generations under filtering and non-filtering conditions*

Quality control analysis

Fecundity

The fecundity was defined as the average number of eggs per female for each strain.

Table 02. Average number of eggs/female for each strain.

Average number of eggs/female for each strain

Strain	Eggs/female	Standard Error
White-eye GSS	71.22	6.55
White-eye GSS/Inv35	83.52	5.81

Generalized Linear Model

group1	group2	p_value	signif
WGSS	WGSS-35	2.24e-01	ns
WGSS	WGSS-35	4.51e-01	ns

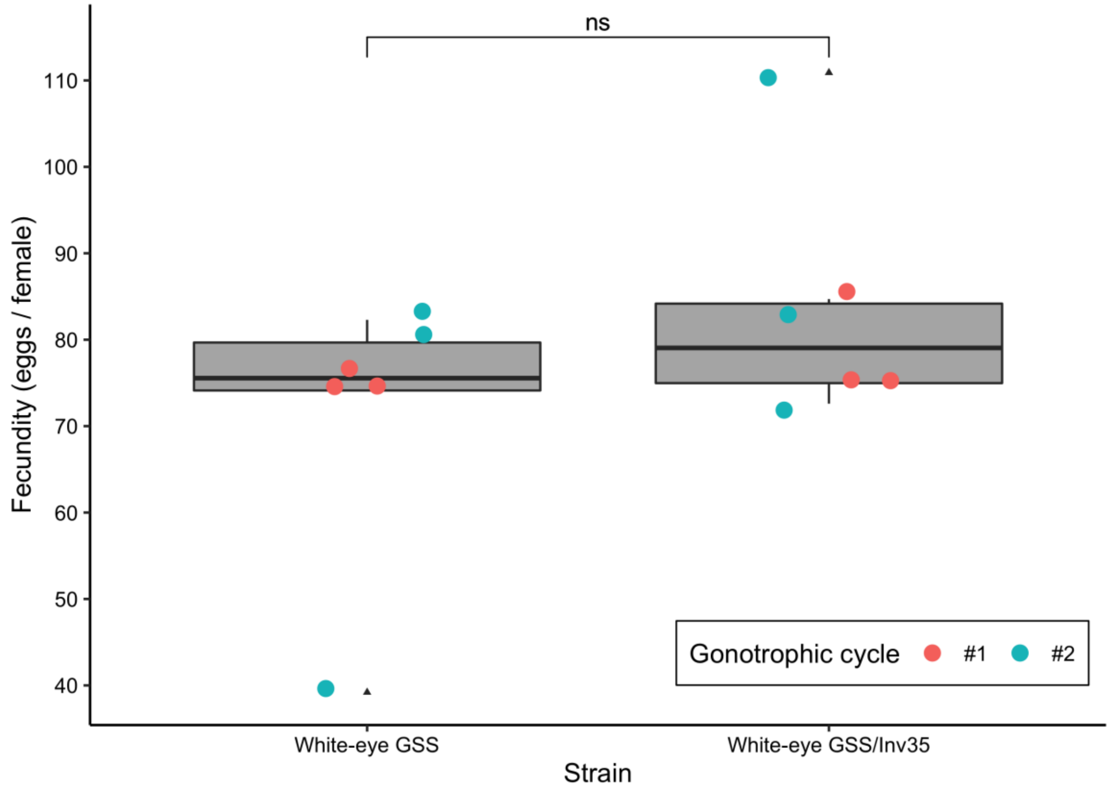

Figure 2. *Fecundity (number of eggs/female) of the White-eye GSS and the White-eye GSS/Inv35 strains per gonotrophic cycle*

Fertility

The fertility was defined as the mean hatch rate for each strain in each gonotrophic cycle.

Table 03. Mean hatch rate (%) for each strain

Mean Hatch Rate (%) for each strain

Strain	Hatch rate (%)	Standard error
White-eye GSS	79.31	4.44
White-eye GSS/Inv35	66.59	3.75

Generalized Linear Model

```
##              Df Sum Sq Mean Sq F value Pr(>F)  
## strain        1  24.99  24.990    4.04 0.0793 .
## GC            1   2.35   2.353    0.38 0.5546  
## strain:GC     1   0.68   0.679    0.11 0.7488  
## Residuals     8  49.49   6.186                 
## ---
## Signif. codes:  0 '***' 0.001 '**' 0.01 '*' 0.05 '.' 0.1 ' ' 1
```

group1	group2	p_value	signif
WGSS-35	WGSS	0.00e+00	***
WGSS-35	WGSS	2.06e-01	ns

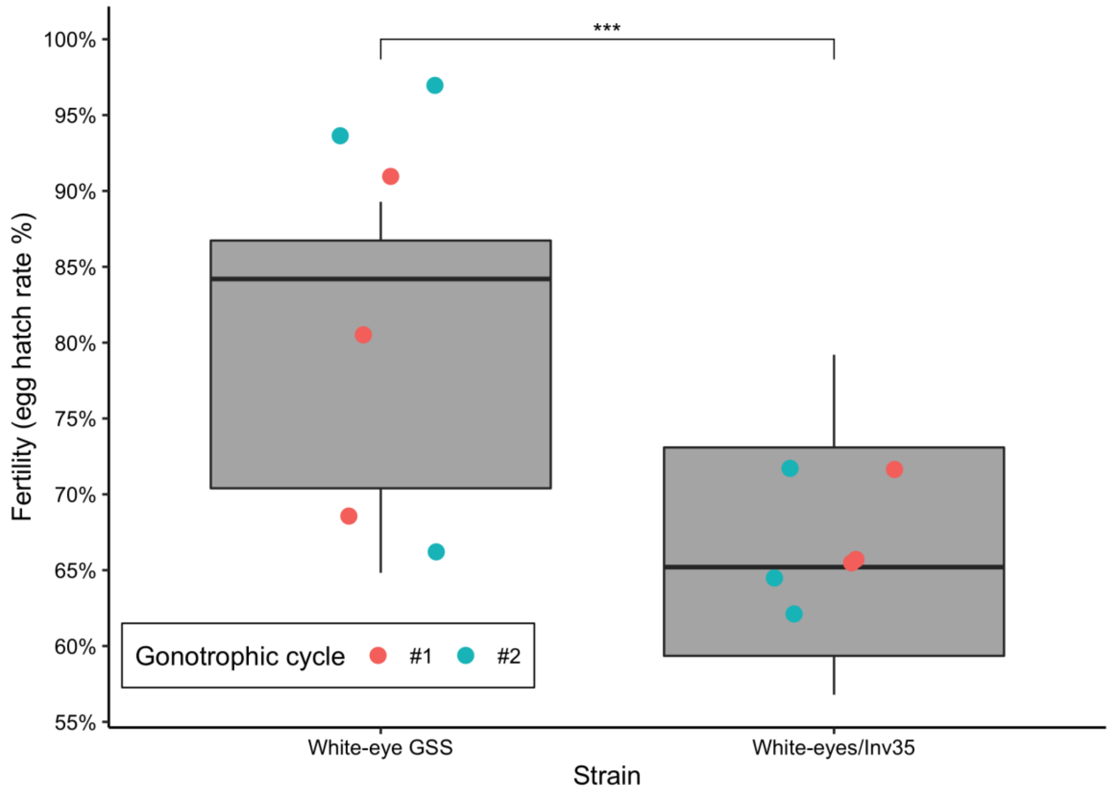

Figure 3. *Fertility (100% × no. L1 larvae/total no. eggs) of the White-eye GSS and the White-eye GSS/Inv35 strains per gonotrophic cycle*

Pupa and adult recovery rates

Recovery was defined as the percentage of individuals reaching a desired stage (pupa or adult), from a known initial number of eggs.

Table 04. Summary of the Pupa and Adult Recovery for Red-eye GSS-PAK, Red-eye GSS/Inv35-PAK, and PAK strains.

Strain	Stage	Average Recovery	Standard Error
White-eye GSS	pupa	0.68	0.08
White-eye GSS	adult	0.65	0.08
White-eye GSS/Inv35	pupa	0.45	0.09
White-eye GSS/Inv35	adult	0.44	0.08

Table 05. Summary of the Female and Male Recovery for Red-eye GSS-PAK, Red-eye GSS/Inv35-PAK, and PAK Strains during pupa stage

Strain	Sex	Average Recovery	Standard Error
White-eye GSS	female	0.32	0.05
White-eye GSS	male	0.36	0.04

Strain	Sex	Average Recovery	Standard Error
White-eye GSS/Inv35	female	0.21	0.03
White-eye GSS/Inv35	male	0.24	0.06

Generalized Linear Model

```
## # A tibble: 2 × 2
##   strain   mean
##   <fct>   <dbl>
## 1 WGSS    0.535
## 2 WGSS-35 0.518

##               Df   Sum Sq   Mean Sq F value Pr(>F)
## strain         1 0.000415 0.0004155    0.28  0.625
## Residuals      4 0.005932 0.0014831
```

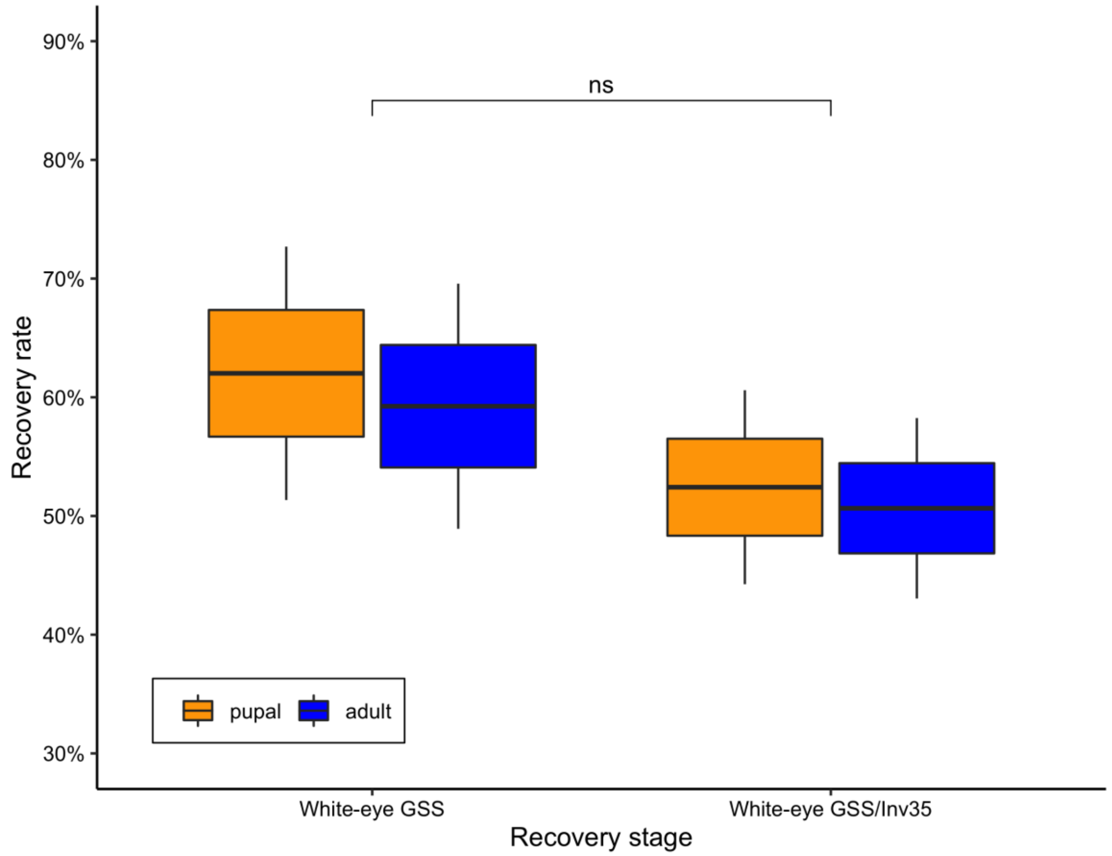

Figure 4. *Recovery rates during different developmental stages for the White-eye GSS and the White-eye GSS/Inv35 strains*

Pupation curve

Pupation curve was defined as the time required from egg hatching to pupation.

Generalized Linear Model

```
.
```

sex	day	WGSS	WGSS-35	diff
female	4 days	0.00	0.00	0.00
female	5 days	0.37	1.46	-1.09
female	6 days	51.47	89.38	-37.90
female	7 days	37.13	5.62	31.51
female	8 days	8.82	0.67	8.15
female	9 days	1.64	1.25	0.39
male	4 days	0.00	0.00	0.00
male	5 days	86.76	69.33	17.43
male	6 days	9.76	25.60	-15.84
male	7 days	3.48	3.47	0.02
male	8 days	0.32	1.33	-1.01
male	9 days	0.00	0.83	-0.83
female	10 days	NA	0.34	NA
male	10 days	NA	0.58	NA

```
##                Df Sum Sq Mean Sq F value Pr(>F)
## strain          1  0.013  0.0129   0.171 0.6802
## sex             1  0.000  0.0000   0.000 1.0000
## day             1  0.336  0.3356   4.468 0.0383 *
## strain:sex      1  0.000  0.0000   0.000 1.0000
## strain:day      1  0.011  0.0114   0.152 0.6982
## sex:day         1  0.157  0.1572   2.093 0.1528
## strain:sex:day  1  0.066  0.0657   0.875 0.3530
## Residuals      66  4.958  0.0751
## ---
## Signif. codes:  0 '***' 0.001 '**' 0.01 '*' 0.05 '.' 0.1 ' ' 1

##
## Call:  glm(formula = percent ~ strain * sex * day, data = pupation_data)
##
## Coefficients:
##         (Intercept)              strain.L               sexmale
##            0.209145              0.208278              0.382272
##                 day      strain.L:sexmale          strain.L:day
##           -0.006048             -0.296729             -0.034064
##         sexmale:day  strain.L:sexmale:day
##           -0.059188              0.048100
##
## Degrees of Freedom: 73 Total (i.e. Null);  66 Residual
## Null Deviance:       5.54
## Residual Deviance: 4.958     AIC: 27.97
```

```
##                   Df Sum Sq Mean Sq F value Pr(>F)
## strain             1  0.013  0.0129   0.171 0.6802
## sex                1  0.000  0.0000   0.000 1.0000
## day                1  0.336  0.3356   4.468 0.0383 *
## strain:sex         1  0.000  0.0000   0.000 1.0000
## strain:day         1  0.011  0.0114   0.152 0.6982
## sex:day            1  0.157  0.1572   2.093 0.1528
## strain:sex:day     1  0.066  0.0657   0.875 0.3530
## Residuals         66  4.958  0.0751
## ---
## Signif. codes:  0 '***' 0.001 '**' 0.01 '*' 0.05 '.' 0.1 ' ' 1
```

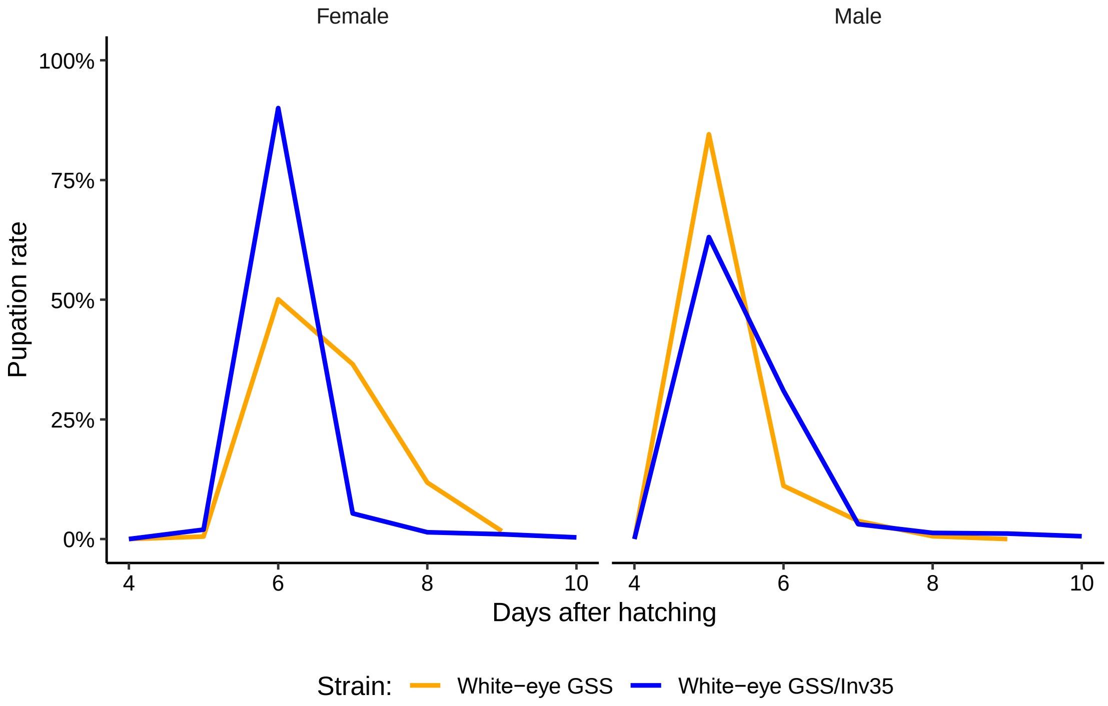

Figure 5. *Pupation curves of females and males of White-eye GSS and White-eye GSS/Inv35 strains*

Pupal Weight

The mean pupal weight obtained from 10 pupae for each sex and strain.

Table 06. Average pupal weight (mg) and standard error for the White-eye GSS and White-eye GSS/Inv35

Strain	Sex	Weight (mg)	Standard Error
WGSS	female	40.14	0.731
WGSS	male	23.38	0.235
WGSS-35	female	44.70	0.674

Strain	Sex	Weight (mg)	Standard Error
WGSS-35	male	26.10	0.277

Generalized Linear Model

```
##             Df    Sum Sq   Mean Sq F value   Pr(>F)    
## strain       1 0.0000662 0.0000662   47.28 3.73e-06 ***
## sex          1 0.0015629 0.0015629 1115.37 3.16e-16 ***
## strain:sex   1 0.0000042 0.0000042    3.02    0.101    
## Residuals   16 0.0000224 0.0000014                     
## ---
## Signif. codes:  0 '***' 0.001 '**' 0.01 '*' 0.05 '.' 0.1 ' ' 1
```

```
## 
## Call:  glm(formula = weight ~ strain * sex, family = poisson(link = "log")
,
##     data = weight)
## 
## Coefficients:
##    (Intercept)        strain.L           sex.L  strain.L:sex.L  
##      -3.431215        0.076952       -0.381318        0.001227  
## 
## Degrees of Freedom: 19 Total (i.e. Null);  16 Residual
## Null Deviance:       0.04965 
## Residual Deviance: 0.000575  AIC: Inf
```

```
##             Df    Sum Sq   Mean Sq F value   Pr(>F)    
## strain       1 0.0000662 0.0000662   47.28 3.73e-06 ***
## sex          1 0.0015629 0.0015629 1115.37 3.16e-16 ***
## strain:sex   1 0.0000042 0.0000042    3.02    0.101    
## Residuals   16 0.0000224 0.0000014                     
## ---
## Signif. codes:  0 '***' 0.001 '**' 0.01 '*' 0.05 '.' 0.1 ' ' 1
```

```
## 
##   Simultaneous Tests for General Linear Hypotheses
## 
## Multiple Comparisons of Means: Tukey Contrasts
## 
## 
## Fit: glm(formula = weight ~ strain * sex, family = poisson(link = "log"), 
##     data = weight)
## 
## Linear Hypotheses:
##                     Estimate Std. Error z value Pr(>|z|)
## WGSS-35 - WGSS == 0   0.1088     2.5335   0.043    0.966
## (Adjusted p values reported -- single-step method)
```

```
## 
## Call:  glm(formula = weight ~ strain, family = poisson(link = "log"),
```

```
##     data = weight %>% filter(sex == "male"))
##
## Coefficients:
## (Intercept)     strain.L
##    -3.70085      0.07782
##
## Degrees of Freedom: 9 Total (i.e. Null);  8 Residual
## Null Deviance:       0.0008545
## Residual Deviance: 0.0001065     AIC: Inf
```

```
##             Df    Sum Sq  Mean Sq F value   Pr(>F)
## strain       1 1.850e-05 1.85e-05   55.88 7.09e-05 ***
## Residuals    8 2.648e-06 3.31e-07
## ---
## Signif. codes:  0 '***' 0.001 '**' 0.01 '*' 0.05 '.' 0.1 ' ' 1
```

```
##
##   Simultaneous Tests for General Linear Hypotheses
##
## Multiple Comparisons of Means: Tukey Contrasts
##
##
## Fit: glm(formula = weight ~ strain, family = poisson(link = "log"),
##     data = weight %>% filter(sex == "male"))
##
## Linear Hypotheses:
##                      Estimate Std. Error z value Pr(>|z|)
## WGSS-35 - WGSS == 0   0.1101     4.0271   0.027    0.978
## (Adjusted p values reported -- single-step method)
```

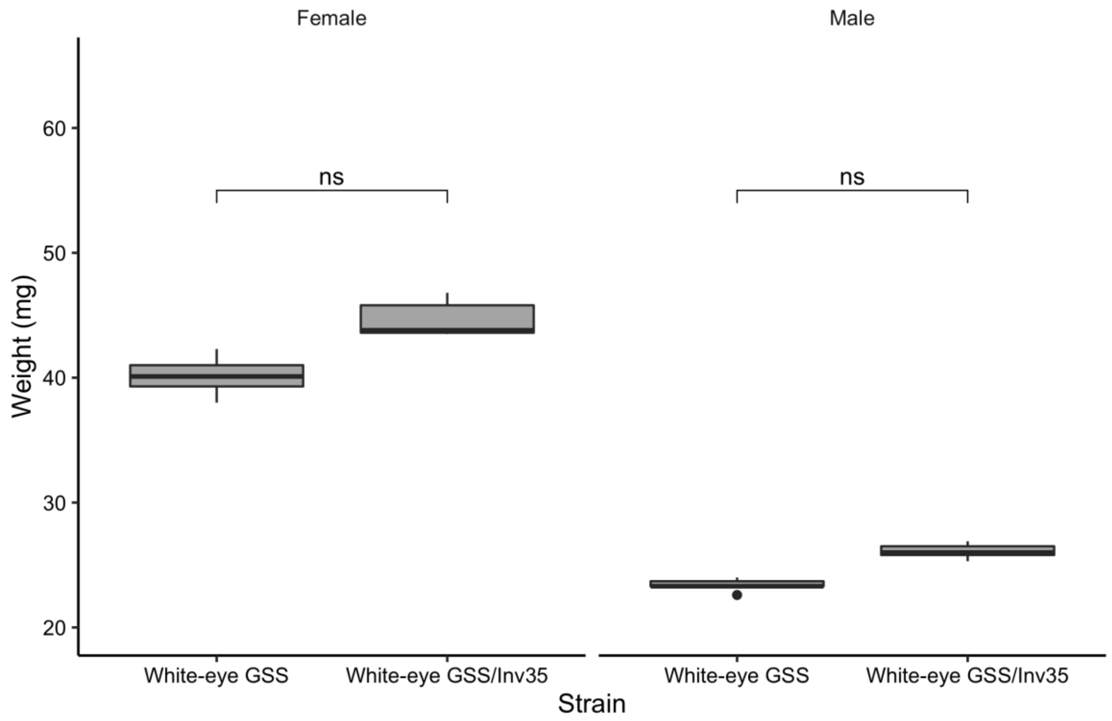

Figure 6. *Weight per 10 male and female pupae of White-eye GSS* and *White-eyes GSS/Inv35* strains

Survival rate

Males

```
## Call:
## survdiff(formula = Surv(time, status) ~ strain, data = male_survival_curve
)
##
##                   N Observed Expected (O-E)^2/E (O-E)^2/V
## strain=WGSS     150       11     13.7     0.518      1.06
## strain=WGSS-35 150       16     13.3     0.531      1.06
##
##  Chisq= 1.1  on 1 degrees of freedom, p= 0.3
```

```
## Call:
## coxph(formula = Surv(time, status) ~ strain, data = male_survival_curve)
##
##   n= 300, number of events= 27
##
##             coef exp(coef) se(coef)     z Pr(>|z|)
## strain.L 0.2823    1.3262   0.2770 1.019    0.308
##
##           exp(coef) exp(-coef) lower .95 upper .95
## strain.L     1.326      0.754    0.7707     2.282
##
```

```
## Concordance= 0.551  (se = 0.048 )
## Likelihood ratio test= 1.06  on 1 df,   p=0.3
## Wald test            = 1.04  on 1 df,   p=0.3
## Score (logrank) test = 1.05  on 1 df,   p=0.3
```

Females

```
## Call:
## survdiff(formula = Surv(time, status) ~ strain, data = female_survival_cur
ve)
##
##                    N Observed Expected (O-E)^2/E (O-E)^2/V
## strain=WGSS    150       47     37.8      2.24      4.44
## strain=WGSS-35 150       31     40.2      2.11      4.44
##
##  Chisq= 4.4  on 1 degrees of freedom, p= 0.04
```

```
## Call:
## coxph(formula = Surv(time, status) ~ strain, data = female_survival_curve)
##
##   n= 300, number of events= 78
##
##             coef exp(coef) se(coef)      z Pr(>|z|)
## strain.L -0.3424    0.7100   0.1637 -2.092   0.0364 *
## ---
## Signif. codes:  0 '***' 0.001 '**' 0.01 '*' 0.05 '.' 0.1 ' ' 1
##
##          exp(coef) exp(-coef) lower .95 upper .95
## strain.L      0.71      1.408    0.5152    0.9786
##
## Concordance= 0.56  (se = 0.028 )
## Likelihood ratio test= 4.48  on 1 df,   p=0.03
## Wald test            = 4.38  on 1 df,   p=0.04
## Score (logrank) test = 4.46  on 1 df,   p=0.03
```

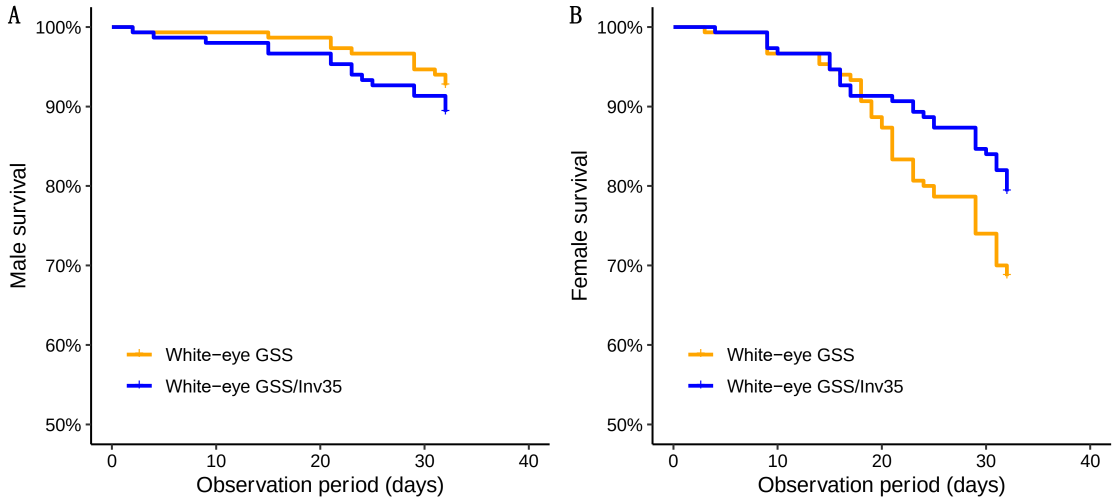

Figure 7. *Survival rate of White-eye GSS and White-eye GSS/Inv35 males (A) and females (B)*

Flight Ability Test

Table 07. Percentage of flyers for non-irradiated White-eye GSS and White-eye GSS/Inv35.

Strain	Percentage of Flyers	Standard Error
White-eye GSS	0.54	0.04
White-eye GSS/Inv35	0.75	0.02

Generalized Linear Model - Flight Ability

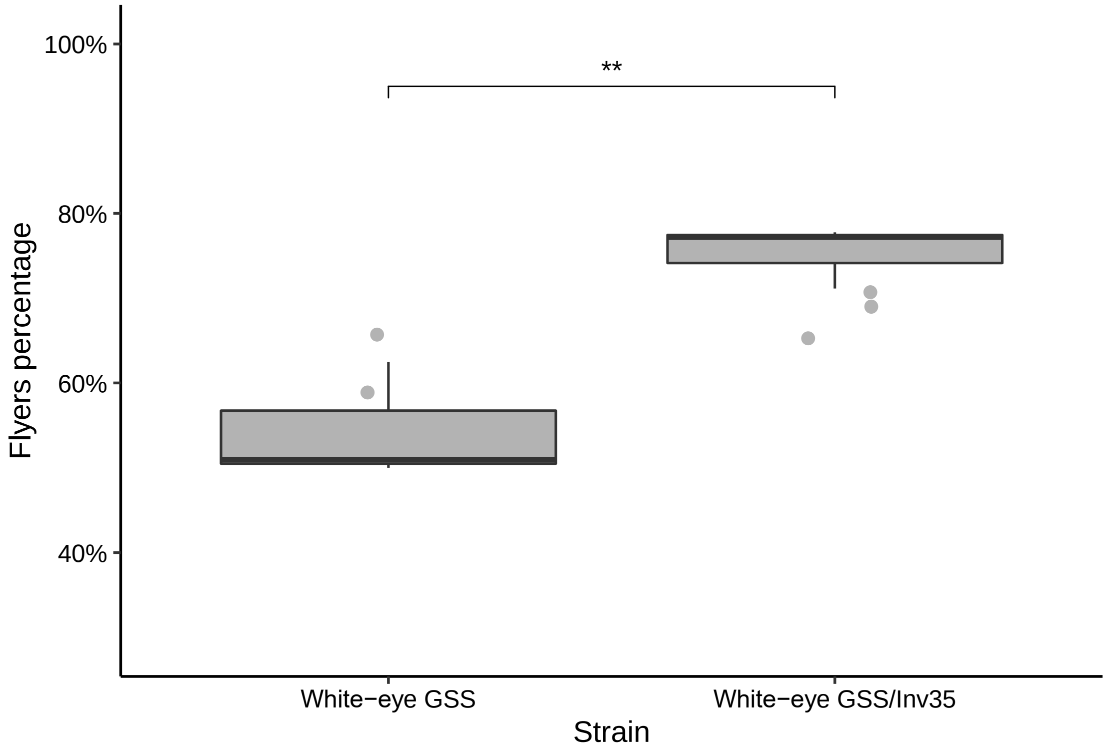

Figure 8. *Flight ability of males of the White-eye GSS and White-eye GSS/Inv35 strains*

R Session Information

```
print(version)

##                _
## platform       x86_64-apple-darwin17.0
## arch           x86_64
## os             darwin17.0
## system         x86_64, darwin17.0
## status
## major          4
## minor          2.0
## year           2022
## month          04
```

```
## day            22
## svn rev        82229
## language       R
## version.string R version 4.2.0 (2022-04-22)
## nickname       Vigorous Calisthenics
```

```
print(sessionInfo())
```

```
## R version 4.2.0 (2022-04-22)
## Platform: x86_64-apple-darwin17.0 (64-bit)
## Running under: macOS Big Sur/Monterey 10.16
##
## Matrix products: default
## BLAS:   /Library/Frameworks/R.framework/Versions/4.2/Resources/lib/libRbla
s.0.dylib
## LAPACK: /Library/Frameworks/R.framework/Versions/4.2/Resources/lib/libRlap
ack.dylib
##
## locale:
## [1] en_US.UTF-8/en_US.UTF-8/en_US.UTF-8/C/en_US.UTF-8/en_US.UTF-8
##
## attached base packages:
## [1] stats     graphics  grDevices utils     datasets  methods   base
##
## other attached packages:
##  [1] lubridate_1.8.0        rcompanion_2.4.15       ggplotify_0.1.0
##  [4] knitr_1.38             DescTools_0.99.45       multcomp_1.4-19
##  [7] TH.data_1.1-1          survival_3.3-1          mvtnorm_1.1-3
## [10] ggstatsplot_0.9.1      survminer_0.4.9         MASS_7.3-56
## [13] ResourceSelection_0.3-5ggfortify_0.4.14        scales_1.2.0
## [16] ggpubr_0.4.0           cowplot_1.1.1           ggsignif_0.6.3
## [19] forcats_0.5.1          stringr_1.4.0           dplyr_1.0.9
## [22] purrr_0.3.4            readr_2.1.2             tidyr_1.2.0
## [25] tibble_3.1.7           ggplot2_3.3.6           tidyverse_1.3.1
##
## loaded via a namespace (and not attached):
##   [1] colorspace_2.0-3       modeltools_0.2-23      ellipsis_0.3.2
##   [4] class_7.3-20           estimability_1.3       parameters_0.17.0
##   [7] fs_1.5.2               gld_2.6.4              mc2d_0.1-21
##  [10] rstudioapi_0.13        proxy_0.4-26           farver_2.1.0
##  [13] fansi_1.0.3            coin_1.4-2             xml2_1.3.3
##  [16] codetools_0.2-18       splines_4.2.0          rootSolve_1.8.2.3
##  [19] libcoin_1.0-9          zeallot_0.1.0          jsonlite_1.8.0
##  [22] broom_0.8.0            km.ci_0.5-6            dbplyr_2.1.1
##  [25] compiler_4.2.0         httr_1.4.2             emmeans_1.7.4-1
##  [28] backports_1.4.1        assertthat_0.2.1       Matrix_1.4-1
##  [31] fastmap_1.1.0          cli_3.3.0              htmltools_0.5.2
##  [34] tools_4.2.0            coda_0.19-4            gtable_0.3.0
##  [37] glue_1.6.2             lmom_2.8               Rcpp_1.0.8.3
##  [40] carData_3.0-5          cellranger_1.1.0       vctrs_0.4.1
```

```
##  [43] nlme_3.1-157             lmtest_0.9-40            insight_0.17.0
##  [46] xfun_0.30                rvest_1.0.2              lifecycle_1.0.1
##  [49] rstatix_0.7.0            zoo_1.8-9                hms_1.1.1
##  [52] parallel_4.2.0           sandwich_3.0-1           expm_0.999-6
##  [55] rematch2_2.1.2           Exact_3.1                yaml_2.3.5
##  [58] gridExtra_2.3            KMsurv_0.1-5             yulab.utils_0.0.4
##  [61] reshape_0.8.9            stringi_1.7.6            highr_0.9
##  [64] paletteer_1.4.0          bayestestR_0.12.1        nortest_1.0-4
##  [67] e1071_1.7-9              boot_1.3-28              matrixStats_0.62.0
##  [70] rlang_1.0.2              pkgconfig_2.0.3          evaluate_0.15
##  [73] lattice_0.20-45          labeling_0.4.2           patchwork_1.1.1
##  [76] tidyselect_1.1.2         plyr_1.8.7               magrittr_2.0.3
##  [79] R6_2.5.1                 multcompView_0.1-8       generics_0.1.2
##  [82] DBI_1.1.2                mgcv_1.8-40              pillar_1.7.0
##  [85] haven_2.5.0              withr_2.5.0              datawizard_0.4.0
##  [88] abind_1.4-5              performance_0.9.0        modelr_0.1.8
##  [91] crayon_1.5.1             car_3.0-12               WRS2_1.1-3
##  [94] survMisc_0.5.6           utf8_1.2.2               correlation_0.8.0
##  [97] tzdb_0.3.0               rmarkdown_2.14           grid_4.2.0
## [100] readxl_1.4.0             data.table_1.14.2        reprex_2.0.1
## [103] digest_0.6.29            xtable_1.8-4             gridGraphics_0.5-1
## [106] statsExpressions_1.3.1 stats4_4.2.0             munsell_0.5.0
```
